# Combined Toxicity Evaluation of Ochratoxin A and Aflatoxin B1 on Kidney and Liver Injury, Immune Inflammation, and Gut Microbiota Alteration Through Pair-Feeding Pullet Model

**DOI:** 10.3389/fimmu.2022.920147

**Published:** 2022-07-29

**Authors:** Hanrui Qing, Shimeng Huang, Kai Zhan, Lihong Zhao, Jianyun Zhang, Cheng Ji, Qiugang Ma

**Affiliations:** ^1^ State Key Laboratory of Animal Nutrition, College of Animal Science and Technology, China Agricultural University, Beijing, China; ^2^ Anhui Province Key Laboratory of Livestock and Poultry Product Safety Engineering, Anhui Academy of Agricultural Sciences, Hefei, China

**Keywords:** ochratoxin A, aflatoxin B1, pullets, microbiota, pair-feeding model

## Abstract

Ochratoxin A (OTA) and aflatoxin B1 (AFB1) are often co-contaminated, but their synergistic toxicity in poultry is limitedly described. Furthermore, the traditional *ad libitum* feeding model may fail to distinguish the specific impact of mycotoxins on the biomarkers and the indirect effect of mildew on the palatability of feed. A pair-feeding model was introduced to investigate the specific effect and the indirect effect of the combined toxicity of OTA and AFB1, which were independent and dependent on feed intake, respectively. A total of 180 one-day-old pullets were randomly divided into 3 groups with 6 replicates, and each replicate contained 10 chicks. The control group (Group A) and the pair-feeding group (Group B) received the basal diet without mycotoxin contamination. Group C was administrated with OTA- and AFB1-contaminated feed (101.41 μg/kg of OTA + 20.10 μg/kg of AFB1). The scale of feeding in Group B matched with the feed intake of Group C. The trial lasted 42 days. Compared with the control group, co-contamination of OTA and AFB1 in feed could adversely affect the growth performance (average daily feed intake (ADFI), body weight (BW), average daily weight gain (ADG), feed conversion ratio (FCR), and shank length (SL)), decrease the relative weight of the spleen (*p* < 0.01), and increase the relative weight of the kidney (*p* < 0.01). Moreover, the reduction of feed intake could also adversely affect the growth performance (BW, ADG, and SL), but not as severely as mycotoxins do. Apart from that, OTA and AFB1 also activated the antioxidative and inflammation reactions of chicks, increasing the level of catalase (CAT), reactive oxygen species (ROS), and interleukin-8 (IL-8) while decreasing the level of IL-10 (*p* < 0.01), which was weakly influenced by the feed intake reduction. In addition, OTA and AFB1 induced histopathological changes and apoptosis in the kidney and liver as well as stimulated the growth of pernicious bacteria to cause toxic effects. There were no histopathological changes and apoptosis in the kidney and liver of the pair-feeding group. The combined toxicity of OTA and AFB1 had more severe effects on pullets than merely reducing feed supply. However, the proper reduction of the feed intake could improve pullets’ physical health by enriching the bacteria *Lactobacillus*, *Phascolarctobacterium*, *Bacteroides*, *Parabacteroides*, and *Barnesiella*.

## Introduction

Ochratoxins (OTs) and aflatoxins (AFs) are secondary metabolites mainly produced by *Aspergillus* (produces AFs and OTs) and *Penicillium* (produces OTs) species. OTs are a group of mycotoxins that consists of ochratoxin A (OTA), ochratoxin B (OTB), and ochratoxin C (OTC) (1). AFs contain more than 20 kinds of compounds with aflatoxin B1 (AFB1), aflatoxin B2 (AFB2), aflatoxin G1 (AFG1), and aflatoxin G2 (AFG2) being the most significant (2). Among them, OTA and AFB1 are the most toxic and have hazardous effects on humans and animals through ingestion, inhalation, and skin contact (3,4). There is a huge chance of agricultural products being co-contaminated by OTA and AFB1. In natural conditions, OTA and AFB1 are mostly occurring during storage due to inadequate drying and improper storage of crops (5). OTA and AFB1 commonly contaminate a wide range of agricultural products, which have been widely reported throughout the world, such as cereals, legumes, nuts, spices, beers, wines, milk, and meats (6). A survey reported that 44%, 40%, and 50% of the mixed cereals, maize, and barley samples, respectively, collected from a feed market in Qatar were concurrently contaminated with AFs and OTA (7). The co-occurrence of OTA and AFB1 accounted for 55% of the multi-contaminated Spanish barley samples (8). Scudamore et al. (9) investigated 330 samples of animal feed ingredients in the United Kingdom and found that maize was the most vulnerable material to mycotoxins.

The structure of OTA is similar to that of the amino acid phenylalanine (Phe), so it inhibits a number of enzymes like Phe-tRNA synthetase that use Phe as a substrate, thus leading to an inhibition of protein synthesis. AFB1 is mainly metabolized in the liver to produce AFB1-8,9-exo-epoxide and 8,9-endo-epoxide and then binds to DNA to cause toxic effects (3). AFB1 exposure has been linked to liver toxicity (10), while the target organs of OTA are considered the kidney and liver (11). The most sensitive organ to the toxicity of combined OTA and AFB1 was the kidney (12). Studies have demonstrated that OTA could induce nephrotoxicity, hepatotoxicity, immunotoxicity, teratogenicity, genotoxicity, and carcinogenicity (13–16). As for AFB1, it can lead to hepatocellular carcinoma, teratogenicity, endocrine problems, infertility, immune suppression, and malabsorption of nutrients (8,17,18). In 1993, OTA and AFB1 were classified as Group 2B and Group I carcinogen for humans by the International Agency for Research on Cancer (IARC). Moreover, according to the European Commission recommendation, the maximum tolerable concentration of OTA in poultry complete feed is 100 μg/kg (2006/576/EC), whereas that of AFB1 is 20 μg/kg (2002/32/EC). According to the national standard of the People’s Republic of China, the maximum tolerable concentration of OTA in poultry complete feed is 100 μg/kg, while that of AFB1 is 10 μg/kg (GB13078-2017, China).

In most studies, OTAs and AFs have synergistic or additive interactions in mortality, body weight (BW), feed intake, egg production, embryo abnormalities, and change of lymphoid organs (19). In hens fed with moldy feed (56 μg/kg of OTA + 136 μg/kg of AFB1) for 42 days, their feed intake, BW gain, egg production (%), and egg weight significantly decreased (*p* < 0.05) (20). Likewise, broilers treated with 1 mg/kg of OTA and 0.5 mg/kg of AFB1 in feed for 49 days had significantly lower BW gain and lower feed consumption than the control (*p* < 0.05).

Recently, studies found that young poultry is more sensitive to mycotoxin than adults due to the incomplete development of organs (21). Khan et al. (20) reported the adverse effects caused by OTA and AFB1 co-contaminated feed (56 μg/kg of OTA + 136 μg/kg of AFB1), such as the decrease in the level of total erythrocyte, total leukocyte, hemoglobin, and hematocrit in the serum and the increase in the relative weight of the liver, kidney, heart, and spleen. There were also pathological changes in the liver, kidney, and spleen like enlargement, ecchymotic hemorrhages on the surface, vacuolar degeneration, cellular infiltration, and congestion. According to Tamilmani et al. (22), the levels of serum alanine aminotransferase (ALT) and alkaline phosphatase (ALP) were increased (*p* < 0.05) in broilers fed with OTA- and AFB1-contaminated diet (100 μg/kg of OTA + 100 μg/kg of AFB1). There was also a significant reduction (*p* < 0.05) in serum uric acid and cholesterol levels at the dose of 200 μg/kg of OTA and 100 μg/kg of AFB1 in feed. It is claimed by some researchers that the combination of OTA and AFB1 (2 mg/kg of OTA + 2.5 mg/kg of AFB1) could lead to less apparent hepatic and more severe kidney lesions (12). However, the specific injury in the kidney and liver remained to be comprehensively studied. In addition, the gut is also an important target tissue for OTA and AFB1 in poultry. So far, only a few studies have focused on the effects of the combined toxicity of OTA and AFB1 in pullets focusing on kidney and liver injury as well as the gut microbiota alteration.

In the traditional toxicity evaluation, the *ad libitum* feeding trial model was often used. However, because of the various visual and organoleptic defects such as off-flavors in mildewed feedstuffs (23), the reduction of feed intake is commonly occurring in animals exposed to mycotoxin-contaminated diets (24). Pair-feeding model is a practical method that can balance the feed consumption between different groups to eliminate effects caused by feed intake. For instance, Shen et al. (25) and Lun et al. (26) applied the pair-feeding model to study the toxic effects of vomitoxin (DON) on pigs. Considering that the reduction of feed intake can be a non-toxic factor to influence animals’ toxic responses, a group fed a normal diet with similar feed intake to the group fed a contaminated diet in the pair-feeding model may help to exclude the interference caused by moldy feed organoleptic defects. However, there are few studies that have tried to distinguish the effects caused by the reduction of feed consumption and combined OTA and AFB1.

In this study, a pair-feeding trial model was introduced to evaluate the toxicity effects on performance, the relative weight of organs, biochemical parameters, oxidative and inflammatory responses, histopathology and apoptosis of the kidney and liver, and the composition of gut microbiota in pullets exposed to OTA and AFB1 simultaneously. The overall aim is to distinguish the specific toxicity conducted through target organs and tissues and the indirect adverse impacts caused by the reduction of the feed supply.

## Materials and Methods

### Production of Ochratoxin A and Aflatoxin B1

An *Aspergillus ochraceus* (CGMCC 3.4412) strain was used to produce OTA, and an *Aspergillus flavus* (CGMCC 3.4408) strain was used to produce AFB1 by artificial infection of sterile maize for 15 days at 28°C, and then the maize was dried and smashed. Then the maize powder was analyzed by near-IR spectroscopy (NIRS; Thermo Nicolet Antaris™ II, Thermo Fisher Scientific, Waltham, MA, USA) to rapidly determine the nutrients of the feed (27). The protein and fat content of the contaminated maize were similar to those of the normal maize.

### Extraction and Clean-Up of Mycotoxins in Feed

Levels of OTA, AFB1, zearalenone (ZEN), and DON in maize samples were determined before diet design and then those in diet samples were determined before the animal trial. Mycotoxin extraction from feed was conducted according to the methods of previous studies (28–31). The samples were extracted with acetonitrile-water (60:40, *v/v*), methanol-water (80:20, *v/v*), acetonitrile-water (90:10, *v/v*), and water to obtain OTA, AFB1, ZEN, and DON, respectively. Then, extracted samples were passed through immunoaffinity clean-up columns (OchraTest^WB^, AflaTest^WB^, ZearalaTest*WB*, and DONTest^WB^; VICAM, Watertown, MA, USA) at a rate of 1-2 drops^s−1^ under gentle pressure provided by a vacuum clean-up assembly. The column was washed with 10 ml of water–methanol (90:10, *v*/*v*) and then dried under nitrogen gas (N_2_) for 5 min. Finally, mycotoxins were eluted from the column by passing 2.0 ml of pure methanol.

### High-Performance Liquid Chromatography

The contents of mycotoxins were determined using a high-performance liquid chromatography (HPLC) system (Shimadzu LC-10 AT, Shimadzu, Tokyo, Japan) equipped with an Agilent^®^ Eclipse Plus C18 column (5 μm, 4.6 × 150 nm) and a fluorescence detector (Shimadzu RF-20A, Shimadzu, Tokyo, Japan). Samples were filtered using a 0.22-μm filter, and 20 μl of volume was loaded to the HPLC system. OTA detection used 333 nm and 477 nm as the wavelengths of excitation and emission, respectively. AFB1 detection used 365 nm and 450 nm as the wavelengths of excitation and emission, respectively. ZEN detection used 274 nm and 440 nm as the wavelengths of excitation and emission, respectively. The detection wavelength was 218 nm for DON. The mobile phase consisted of acetonitrile-water-glacial acetic acid (99:99:2, v/v/v) for OTA, acetonitrile-methanol-water (45:45:110, v/v/v) for AFB1, acetonitrile-water-methanol (46:46:8, v/v/v) for ZEN, and methanol-water (30:70, v/v) for DON and the flow rate was 1 mL min−1. The temperature of the column was set at 30°C for OTA, AFB1, ZEN, and DON [49].

### Animal Trial in Pullets

All procedures were reviewed and approved by the Laboratory Animal Welfare and Animal Experimental Ethical Committee of China Agricultural University (No. AW 13301202-1-12). The trial strictly complied with the standard operating procedures for experimental animals of the Ministry of Science and Technology (Beijing, China), and every effort was made to minimize suffering.

A total of 180 one-day-old Jingfen No. 1 commercial chicks were randomly allocated to three feeding treatments of 60 chicks each with 6 replicates per group, and each replicate contained 10 chicks. The nutrient values and feeding procedures referred to the NY/T 33-2004 (China) and the manual for Jingfen No. 1 chicks (Huadu Yukou, Beijing, China). The control group (Group A) and the pair-feeding group (Group B) received the basal diet containing 65% normal maize (OTA, 0 μg/kg diet; AFB1, 0 μg/kg diet), while Group C received 65% contaminated maize (OTA, 101.41 μg/kg diet; AFB1, 20.10 μg/kg diet). The contaminated diet in Group C was produced by replacing the normal maize with contaminated maize on the basis of the basal diet. The composition and nutrient levels of diets are shown in **Supplementary Table 1**.

All groups were supplied with the amount of feed recommended by the commercial layer’s management manual on the first day of the trial. From the second day, the feeding amount in Group A and Group C was still supplied according to this manual, while the amount of feeding in Group B matched with the feed intake of Group C. Feed for the pair-feeding group was supplied once daily in the morning, receiving the amount that calculated according to the proportion of feed intake of Group C and Group A at the previous day. If set “A*_n_*_−1_” and “C*_n_*_−1_” are the measured feed intake of Group A and Group C on the previous day, and set “A*_n_*” is the feeding amount of Group A on the day “*n*” recommended by this management manual, then the feeding amount of Group B on the day “*n*” can be represented as “ $B_{n}=A_{n}\times \frac{C_{n-1}}{A_{n-1}}$ ”. The pair-feeding started on the 2nd day of the trial. The feeding trial lasted for 42 days from the 1st day, and the performance of pullets was calculated from 1 to 42 days. BW and shank length (SL) were measured on the 1st and 42nd days of age. Average daily feed intake (ADFI = (Feeding amount– Leftover feed amount)/days on trial), Average daily weight gain (ADG = Liveweight (end) − Liveweight (initial)/days on trial), and Feed conversion ratio (FCR = ADFI/ADG) were recorded. ADG and FCR were reported for the whole trial period.

### Sample Collection

After the 42-day feeding trial, blood, spleen, kidney, and liver samples were collected. Blood samples were stored at –20°C until biochemical analysis. The spleen, left kidney, and liver were weighed and calculated (relative weight of internal organ = internal organ weight/BW × 100). The kidney and liver samples were transferred into 10% formaldehyde for histopathological analysis, and the kidney and liver tissues were stored at −80°C until biochemical analysis.

### Analysis of Biochemical Parameters in Serum, Kidney, and Liver

The activity of phosphoenolpyruvate carboxykinase (PEPCK) (A131-1-1) as well as the concentrations of ALP (A059-1-1), aspartate aminotransferase (AST) (C010-3-1), ALT (C009-3-1), total antioxidant capacity (T-AOC) (A015-1-2), superoxide dismutase (SOD) (A001-3-2), malondialdehyde (MDA) (A003-1-2), catalase (CAT) (A007-2-1), reactive oxygen species (ROS) (E004-1-1), glutathione (GSH) (A006-1-1), glutathione peroxidase (GSH-Px) (A005-1-2), and oxidized glutathione (GSSG) (A061-2-1) in the serum, kidney, and liver were measured with the assay kits (Nanjing Jiancheng Bioengineering Institute, Nanjing, China). The levels of leucine aminopeptidase (LAP) (H454), alanine aminopeptidase (AAP) (H583), cytokine interleukin-6 (IL-6) (H007-1-2), interleukin-8 (IL-8) (H008), interleukin-10 (IL-10) (H009-1), tumor necrosis factor-α (TNF-α) (H023), and interferon-γ (IFN-γ) (H025) were measured with the ELISA kits (Nanjing Jiancheng Bioengineering Institute, Nanjing, China). All procedures were performed according to the manufacturer’s instructions, and the number of the kit has been given above.

### H&E and TUNEL Staining

Samples from the liver and kidney were fixed and immersed immediately in 4% paraformaldehyde for 24 h and embedded in paraffin by a routine procedure. The H&E was performed according to the conventional histopathologic examination. The *in situ* cell death was detected by the Apop Tag kit (S7101-Kit; Oncor, Gaithersburg, MD, USA) and performed according to the manufacturer’s instructions.

### 16S rRNA Gene Sequencing of the Gut Microbiota

The cecal chyme of pullets was collected and stored at −80°C. The Omega Bio-Tek stool DNA kit (Omega, Norcross, GA, USA) was used to extract the total DNA of the cecal chyme of the pullets. The specific operation was carried out according to the instructions, and the quality and integrity of the extracted DNA were tested by a NanoDrop 2000 spectrophotometer (Thermo Scientific, Waltham, MA, USA). The purified DNA was stored in a −80°C refrigerator for subsequent analysis.

Based on the Illumina HiSeq sequencing platform, according to the characteristics of the 16S region to be amplified, the QIIME (Quantitative Insights into Microbial Ecology) method was used to extract the microbial DNA as a template, with 338F (5′-barcode ACTCCTACGGG AGGCAGCAG-3′) and 806R (5′-GGACTACHVGGGTWTCTAAT-3′) primers for PCR amplification. The PCR products were then evaluated by the AxyPrep DNA gel extraction kit (Axygen Biosciences, Union City, CA, USA). On-board sequencing was performed using HiSeq2500, PE250 (Shanghai MajorBio Biopharma Technology Co., Ltd., Shanghai, China).

### Statistical Analysis

Statistically significant differences between mean values of the parameters tested in the animal feeding trial were analyzed with ANOVA using Tukey’s honestly significant difference (HSD) *post-hoc* test in the SPSS statistical software package (version 22, Armonk, NY, USA). The main effects of feed reduction and mycotoxins specific were analyzed with an independent-samples t-test in the SPSS. The differences were considered statistically significant if the *p*-values <0.05. The GraphPad Prism software for Windows was used to generate graphs (version 900, San Diego, CA, USA). Microbial diversity analysis was performed using the Shanghai Majorbio I-Sanger cloud platform (https://www.i-sanger.com/).

## Results

### Dietary Mycotoxin Concentrations

OTA, AFB1, ZEN, and DON were not detectable in the normal maize and the basal feed. The concentrations of OTA, AFB1, ZEN, and DON in the contaminated maize were 160.40, 32.48, 20.48, and 93.96 μg/kg, respectively. The concentrations of OTA, AFB1, ZEN, and DON in the contaminated feed were 101.41, 20.10, 13.31, and 60.11 μg/kg, respectively. As the levels of OTA, AFB1, ZEN, and DON in the contaminated feed accounted for 101.41%, 201.00%, 2.67%, and 2.00%, respectively, of the maximum tolerable concentrations for poultry feed (OTA, 100 μg/kg; AFB1, 10 μg/kg; ZEN, 500 μg/kg; and DON, 3,000 μg/kg; GB13078-2017, China), the influence of ZEN and DON could be neglected in this trial; thus, the main toxic effects in this trial were caused by OTA and AFB1 in feed.

### Growth Performance

No pullets died during the trial. The effects of combined OTA and AFB1 on pullets’ growth performance are shown in **Table 1**. The ADFI of Group A was significantly higher than that of Group C and Group B (*p* < 0.01), while the ADFI was very similar between Group C and Group B, as we expected. The BW and the ADG of Group C pullets were both significantly lower than those of Group A (*p* < 0.01) and Group B (*p* < 0.01). As for the FCR, it was significantly higher in Group C than in the other groups (*p* < 0.01), while there was no statistical difference between Group A and Group B. Likewise, the SL of Group C was the lowest among the three groups (*p* < 0.01). In addition, the growth performance parameters (BW, ADG, and SL) of Group C were lower than those of Group A and Group B. In other words, the reduction of feed intake adversely affected the growth performance (BW, ADG, and SL), but not as severely as mycotoxins do.

**Table 1:** The effects of combined OTA and AFB1 on the growth performance in pullets (n = 6, mean ± SEM).

Parameter	Period	Group A	Group B	Group C	SEM	*p*-Value	A vs B *p*-Value	B vs C *p*-Value
Average daily feed intake (g/day)	1–42 days	23.06^a^	17.73^b^	17.71^b^	0.395	< 0.01	< 0.01	0.979
Body weight (g)	1 day	40.84	40.83	40.83	0.069	0.98	0.890	1.000
	42 days	409.31^a^	338.29^b^	268.06^c^	4.932	< 0.01	< 0.01	< 0.01
Average daily weight gain (g/day)	1–42 days	9.21^a^	7.44^b^	5.68^c^	0.123	< 0.01	< 0.01	< 0.01
Feed conversion ratio (g/g)	1–42 days	2.51^a^	2.39^a^	3.12^b^	0.080	< 0.01	0.056	< 0.01
Shank length (mm)	1 day	28.35	28.45	28.08	0.344	0.53	0.745	0.298
	42 days	70.46^a^	66.02^b^	58.87^c^	0.850	< 0.01	< 0.01	< 0.01

Groups A, B, and C are the control group, the pair-feeding group, and the OTA+AFB1 group, respectively. A vs B p-value, main effects of feed reduction; B vs C p-value, main effects of toxins specific. Data were analyzed with ANOVA, Tukey’s HSD, and independent-samples t-test.

OTA, ochratoxin A; AFB1, aflatoxin B1; HSD, honestly significant difference.

^a,b,c^Means with different superscripts in the same row differ significantly (p < 0.01).

### The Relative Weight of Organs in Pullets

The effects of combined OTA and AFB1 on the relative weight of organs in pullets are presented in **Table 2**. The relative weight of the spleen in Group C was significantly lower than in the other two groups (*p* < 0.01). Moreover, the relative weight of kidneys in Group C was significantly higher than that in Group A (*p* < 0.01). As for the relative weight of the liver, pullets of Group C had the highest figure among these three groups, although there was no statistically significant difference. Likewise, there was also no statistically significant difference in the viscera relative weight (spleen and kidney) between Group A and Group B, and it is noteworthy that the reduction of the feed intake significantly increased the relative weight of the liver as compared with Group A (*p* < 0.05).

**Table 2:** The effects of combined OTA and AFB1 on the relative weight of organs in pullets (n = 6, mean ± SEM).

Relative weight (g/100 g BW)	Group A	Group B	Group C	SEM	*p*-Value	A vs B *p*-Value	B vs C *p*-Value
Spleen	0.40^a^	0.45^a^	0.27^b^	0.040	<0.01	0.244	<0.01
Kidney (left)	0.43^a^	0.47^a,b^	0.53^b^	0.029	<0.01	0.078	0.091
Liver	2.80	3.13	3.27	0.209	0.09	0.033	0.559

Groups A, B, and C are the control group, the pair-feeding group, and the OTA+AFB1 group, respectively. A vs B p-value, main effects of feed reduction; B vs C p-value, main effects of toxins specific. Data were analyzed with ANOVA, Tukey’s HSD, and independent-samples t-test.

OTA, ochratoxin A; AFB1, aflatoxin B1; BW, body weight; HSD, honestly significant difference.

^a,b^Means with different superscripts in the same row differ significantly (p < 0.01).

### Serum Biochemical Parameters

**Figure 1:**
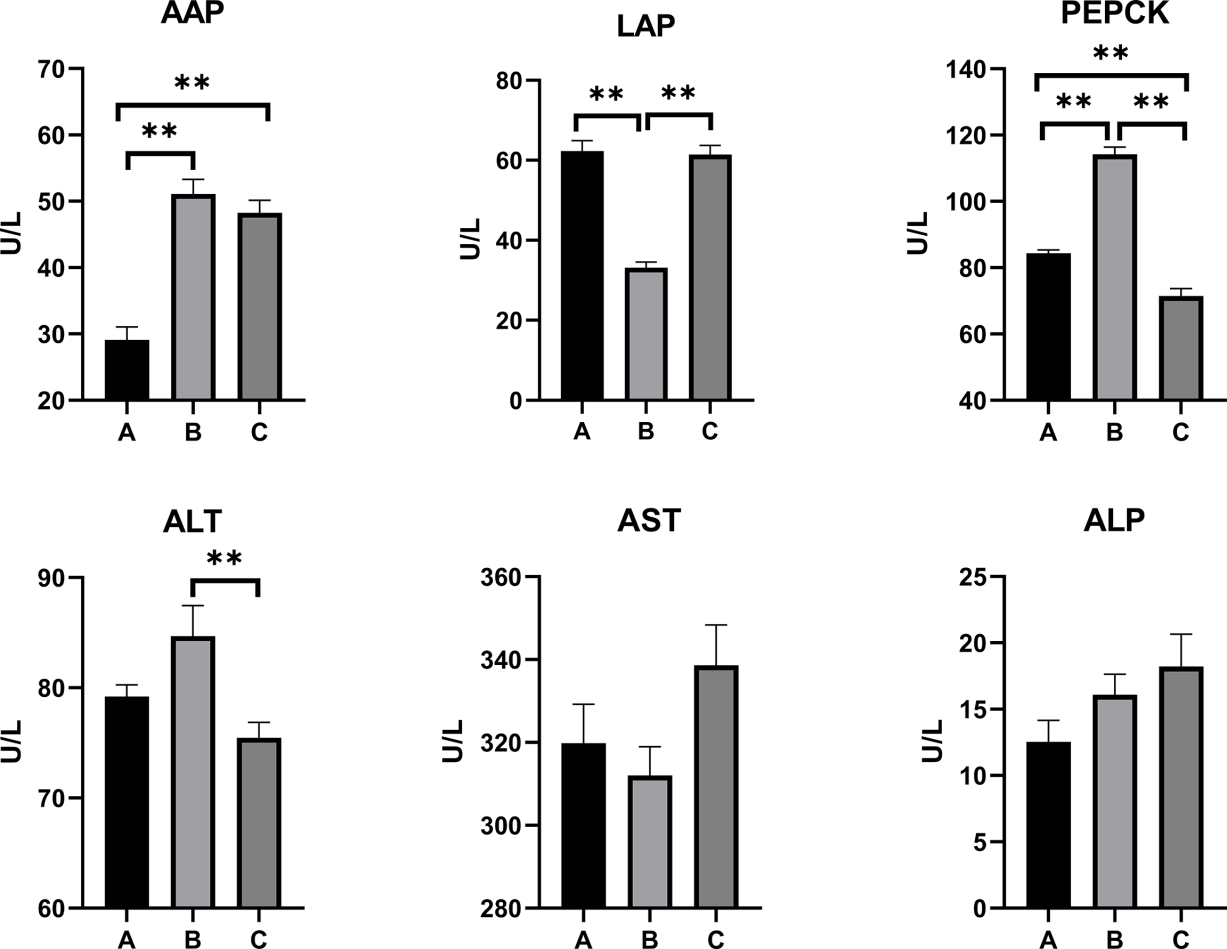
Effects of OTA and AFB1 on serum biochemical parameters of pullets after 42 days (n = 6, mean ± SEM), including AAP, LAP, PEPCK, ALT, AST, and ALP. Groups A, B, and C are the control group, the pair-feeding group, and the OTA+AFB1 group, respectively. Data were analyzed with ANOVA and Tukey’s HSD, and significant differences were defined as **p < 0.01. OTA, ochratoxin A; AFB1, aflatoxin B1; AAP, alanine aminopeptidase; LAP, leucine aminopeptidase; PEPCK, phosphoenolpyruvate carboxykinase; ALT, alanine aminotransferase; AST, aspartate aminotransferase; ALP, alkaline phosphatase; HSD, honestly significant difference.

The levels of the serum biochemical parameters in the pullets kept in different treatment groups for 42 days are shown in **Figure 1**. The concentration of AAP significantly increased in Group C and Group B when compared with Group A (*p* < 0.01). The pullets of Group C had the lowest level of serum PEPCK concentration (*p* < 0.01), while Group B pullets had the highest PEPCK level among the three groups (*p* < 0.01). Moreover, the lowest LAP level was observed in Group B (*p* < 0.01), while there was no statistical difference between Group C and Group A.

As for liver damage parameters, including ALT, AST, and ALP, there were no statistically significant differences between Group C and Group A. However, the concentration of ALT in Group B was significantly higher than in Group C (*p* < 0.01).

### Oxidative Stress and Antioxidant Status in the Serum, Kidney, and Liver

**Figure 2:**
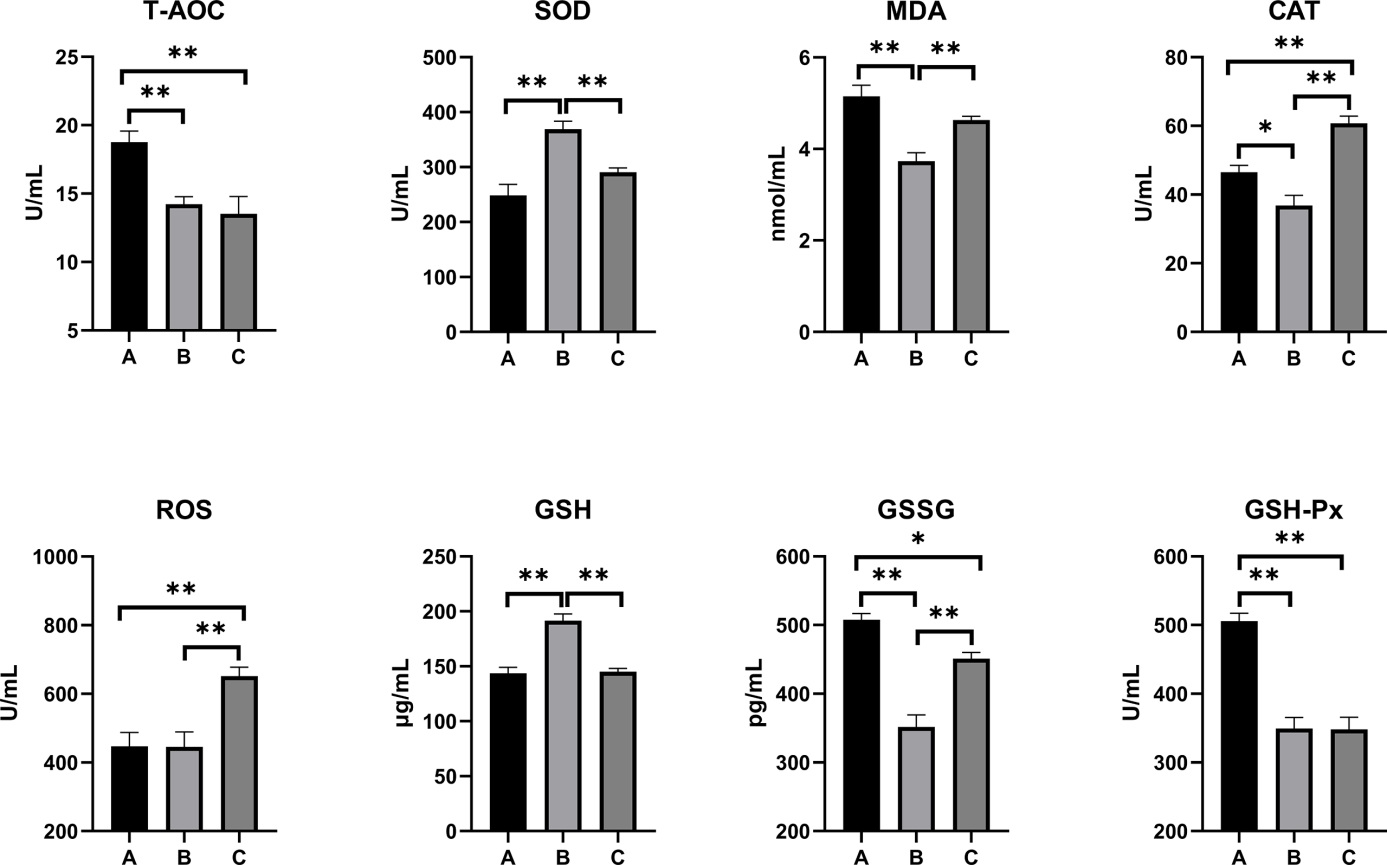
Effects of OTA and AFB1 on serum oxidative stress and antioxidant status of pullets after 42 days (n = 6, mean ± SEM), including T-AOC, SOD, MDA, CAT, ROS, GSH, GSSG, and GSH-Px. Groups A, B, and C are the control group, the pair-feeding group, and the OTA+AFB1 group, respectively. Data were analyzed with ANOVA and Tukey’s HSD, and significant differences were defined as *p < 0.05, and **p < 0.01. OTA, ochratoxin A; AFB1, aflatoxin B1; T-AOC, total antioxidant capacity; SOD, superoxide dismutase; MDA, malondialdehyde; CAT, catalase; ROS, reactive oxygen species; GSH, glutathione; GSSG, oxidized glutathione; GSH-Px, glutathione peroxidase; HSD, honestly significant difference.

**Figure 3:**
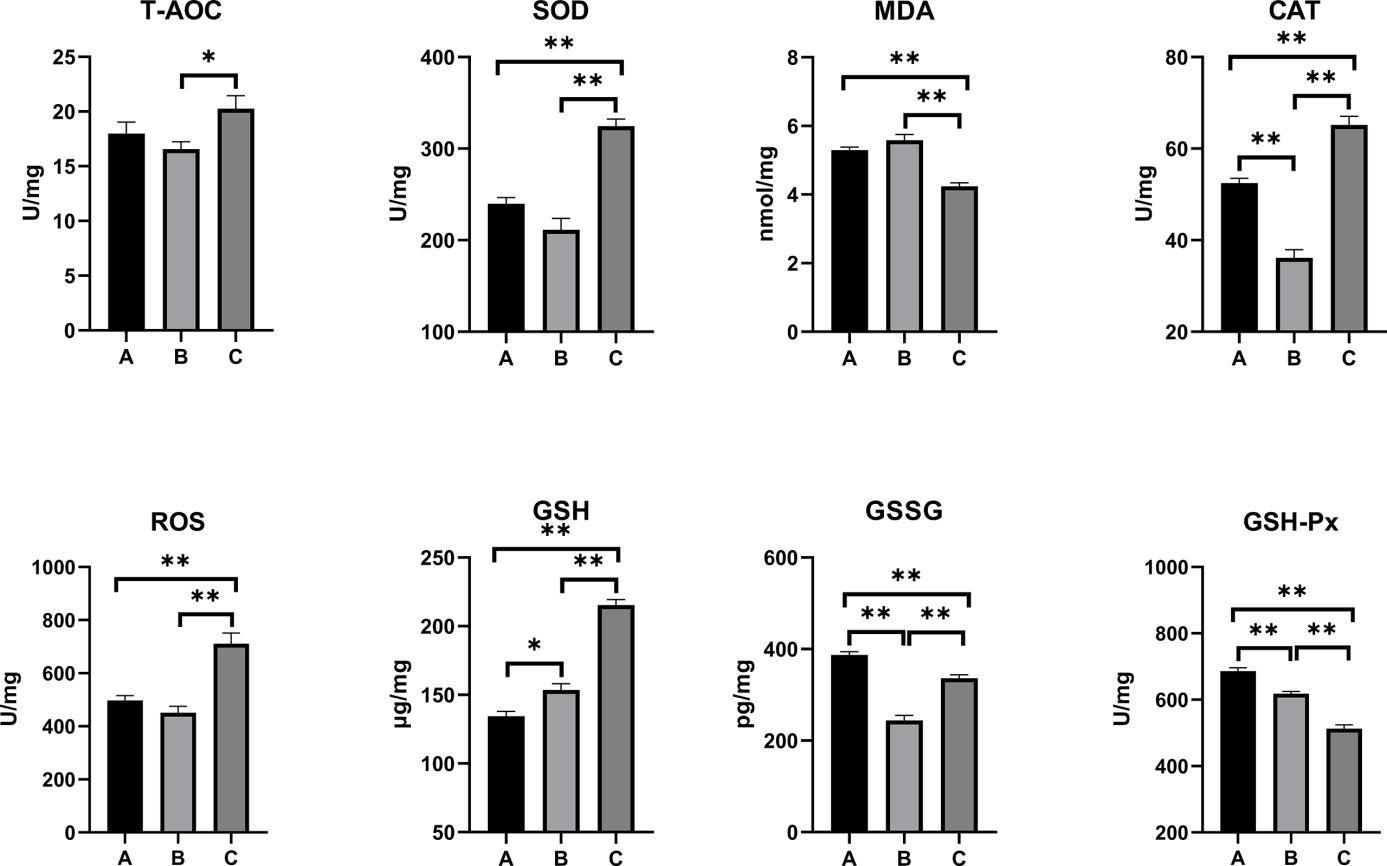
Effects of OTA and AFB1 on kidney oxidative stress and antioxidant status of pullets after 42 days (n = 6, mean ± SEM), including T-AOC, SOD, MDA, CAT, ROS, GSH, GSSG, and GSH-Px. Groups A, B, and C are the control group, the pair-feeding group, and the OTA+AFB1 group, respectively. Data were analyzed with ANOVA and Tukey’s HSD, and significant differences were defined as *p < 0.05 and **p < 0.01. OTA, ochratoxin A; AFB1, aflatoxin B1; T-AOC, total antioxidant capacity; SOD, superoxide dismutase; MDA, malondialdehyde; CAT, catalase; ROS, reactive oxygen species; GSH, glutathione; GSSG, oxidized glutathione; GSH-Px, glutathione peroxidase; HSD, honestly significant difference.

**Figure 4:**
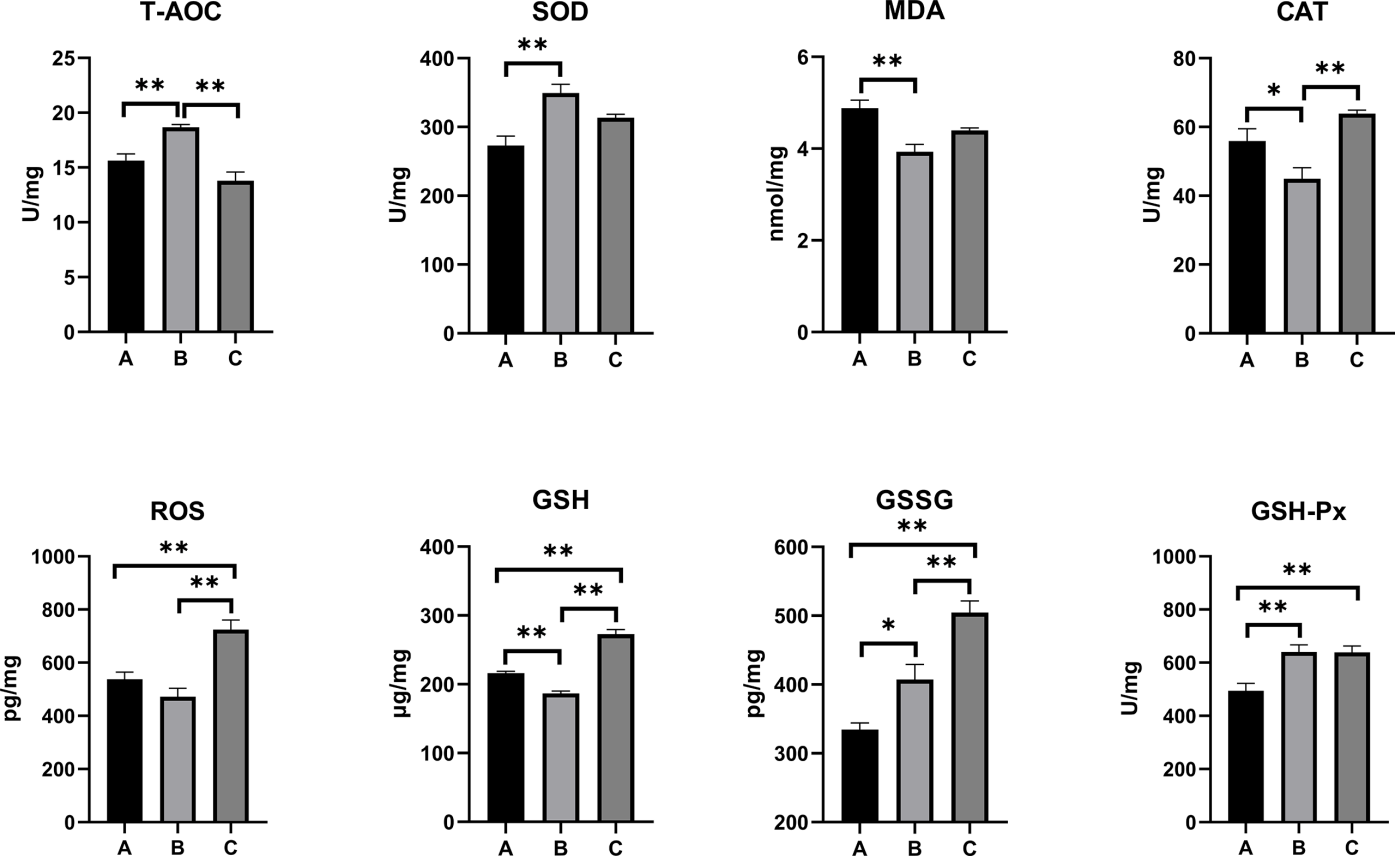
Effects of OTA and AFB1 on liver oxidative stress and antioxidant status of pullets after 42 days (n = 6, mean ± SEM), including T-AOC, SOD, MDA, CAT, ROS, GSH, GSSG, and GSH-Px. Groups A, B, and C are the control group, the pair-feeding group, and the OTA+AFB1 group, respectively. Data were analyzed with ANOVA and Tukey’s HSD, and significant differences were defined as *p < 0.05 and **p < 0.01. OTA, ochratoxin A; AFB1, aflatoxin B1; T-AOC, total antioxidant capacity; SOD, superoxide dismutase; MDA, malondialdehyde; CAT, catalase; ROS, reactive oxygen species; GSH, glutathione; GSSG, oxidized glutathione; GSH-Px, glutathione peroxidase; HSD, honestly significant difference.

The oxidative stress and antioxidant status on the serum, kidney, and liver of combined OTA- and AFB1-exposed pullets have been presented in **Figures 2**–**4**. The levels of serum T-AOC (*p* < 0.01), GSSG (Group B: *p* < 0.01; Group C: *p* < 0.05), and GSH-Px (*p* < 0.01) were significantly lower in Group B and Group C when compared with Group A. Compared with Group A, the levels of serum SOD and GSH increased significantly in Group B (*p* < 0.01), while the levels of serum MDA (*p* < 0.01) and CAT (*p* < 0.05) decreased significantly. The levels of serum CAT and ROS increased significantly in Group C (*p* < 0.01) when compared with other groups. The antioxidative reaction in the kidney was the most active. In Group B and Group C, the level of kidney GSH was significantly higher, and the levels of kidney GSSG and GSH-Px were significantly lower as compared with Group A (*p* < 0.01). The levels of kidney CAT decreased significantly in Group B compared with other groups (*p* < 0.01). In Group C, the concentrations of kidney SOD, CAT, and ROS increased significantly (*p* < 0.01), while the level of MDA decreased significantly (*p* < 0.01) as compared with other groups. As for the concentrations of liver GSSG and GSH-Px, they increased significantly in Group B (GSSG: *p* < 0.05; GSH-Px: *p* < 0.01) and Group C (*p* < 0.01) when compared with Group A. The levels of liver T-AOC and SOD were significantly higher (*p* < 0.01), and the levels of MDA (*p* < 0.01), CAT (*p* < 0.05), and GSH (*p* < 0.01) were significantly lower in Group B when compared with Group A. The levels of ROS and GSH increased significantly in Group C compared with other groups (*p* < 0.01).

### Inflammatory Responses in the Serum, Kidney, and Liver

**Figure 5:**
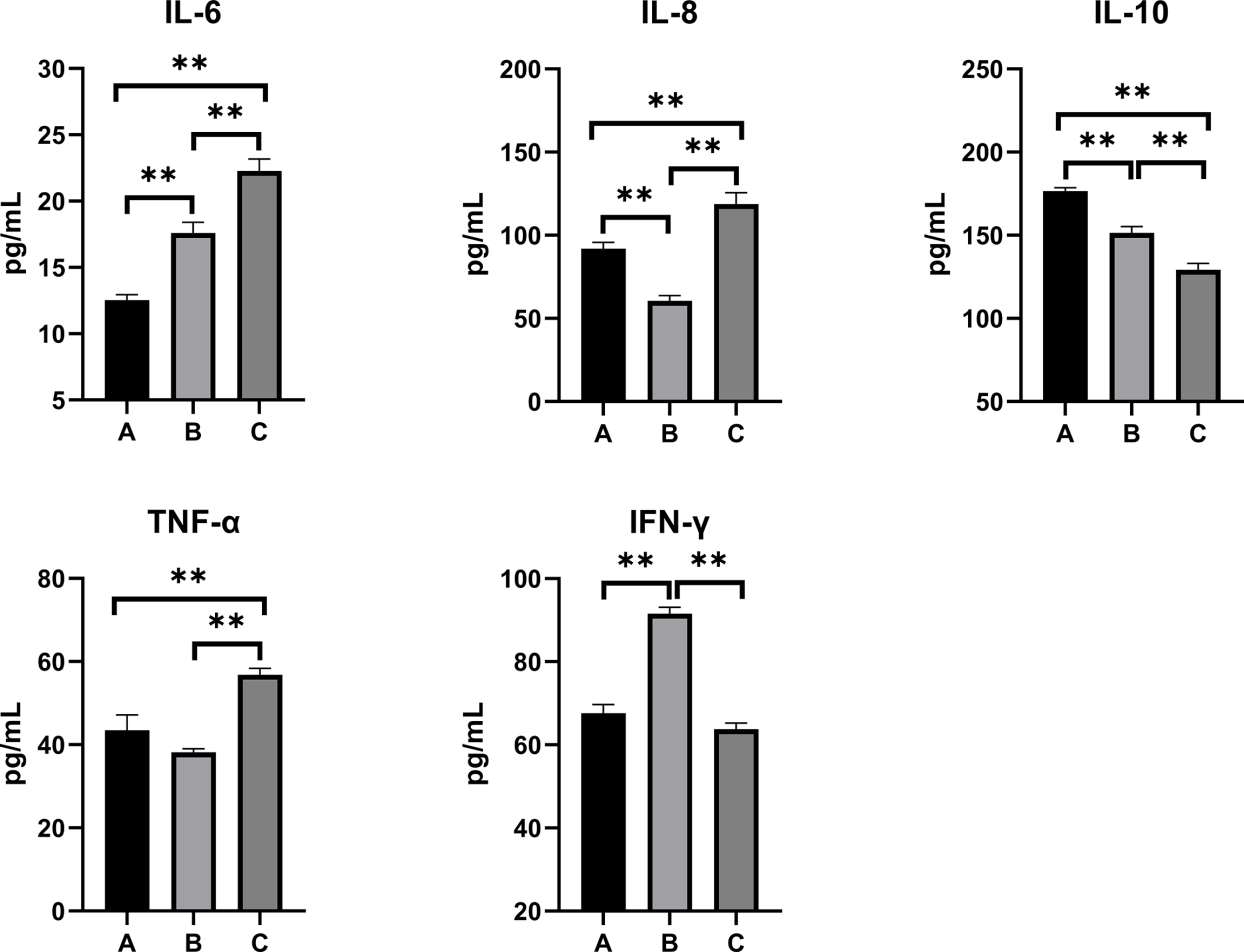
Effects of OTA and AFB1 on serum cytokines (IL-6, IL-8, IL-10, TNF-α, and IFN-γ) of pullets after 42 days (n = 6, mean ± SEM). Groups A, B, and C are the control group, the pair-feeding group, and the OTA+AFB1 group, respectively. Data were analyzed with ANOVA and Tukey’s HSD, and significant differences were defined as **p < 0.01. OTA, ochratoxin A; AFB1, aflatoxin B1; HSD, honestly significant difference.

**Figure 6:**
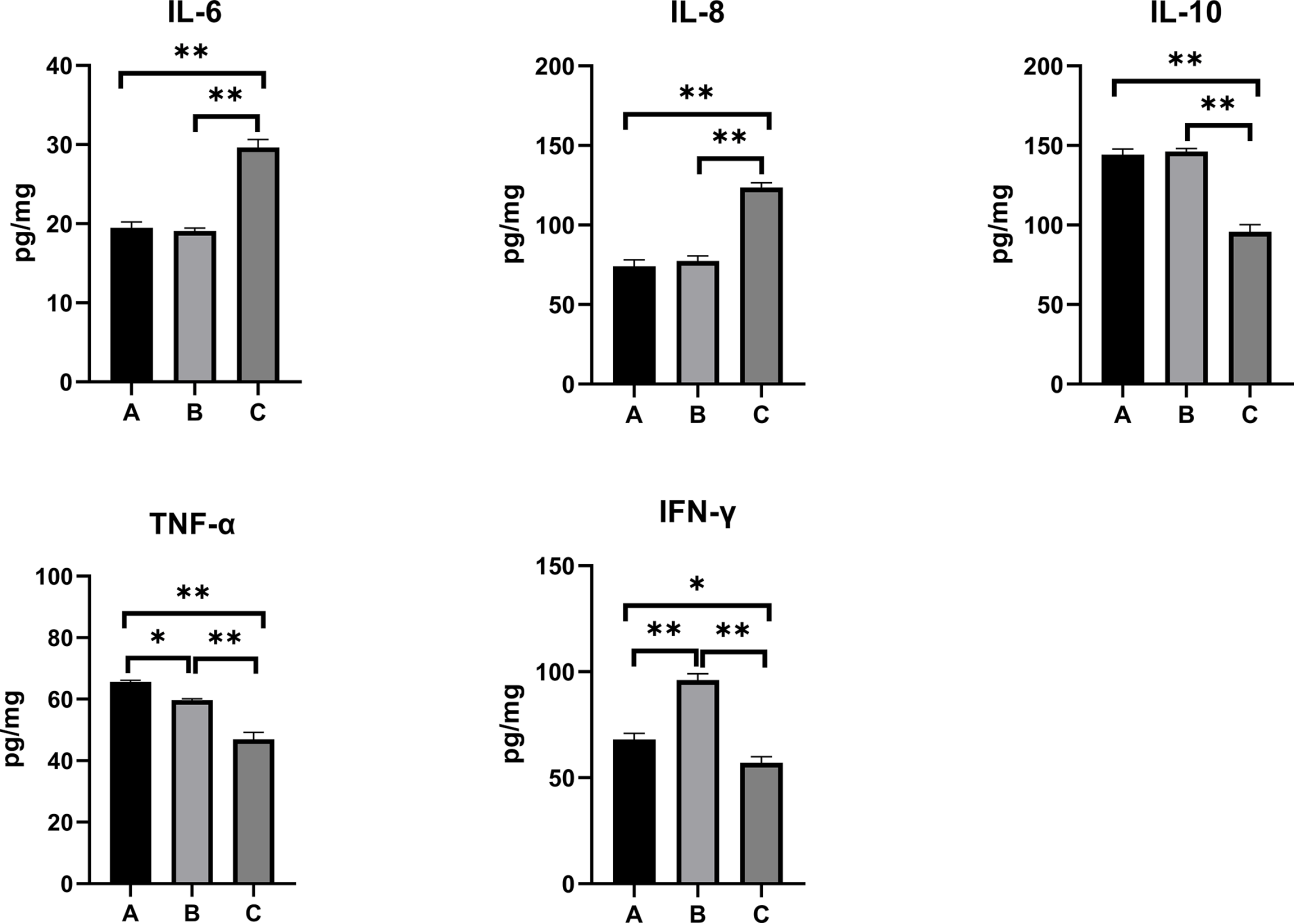
Effects of OTA and AFB1 on kidney cytokines (IL-6, IL-8, IL-10, TNF-α, and IFN-γ) of pullets after 42 days (n = 6, mean ± SEM). Groups A, B, and C are the control group, the pair-feeding group, and the OTA+AFB1 group, respectively. Data were analyzed with ANOVA and Tukey’s HSD, and significant differences were defined as *p < 0.05 and **p < 0.01. OTA, ochratoxin A; AFB1, aflatoxin B1; HSD, honestly significant difference.

**Figure 7:**
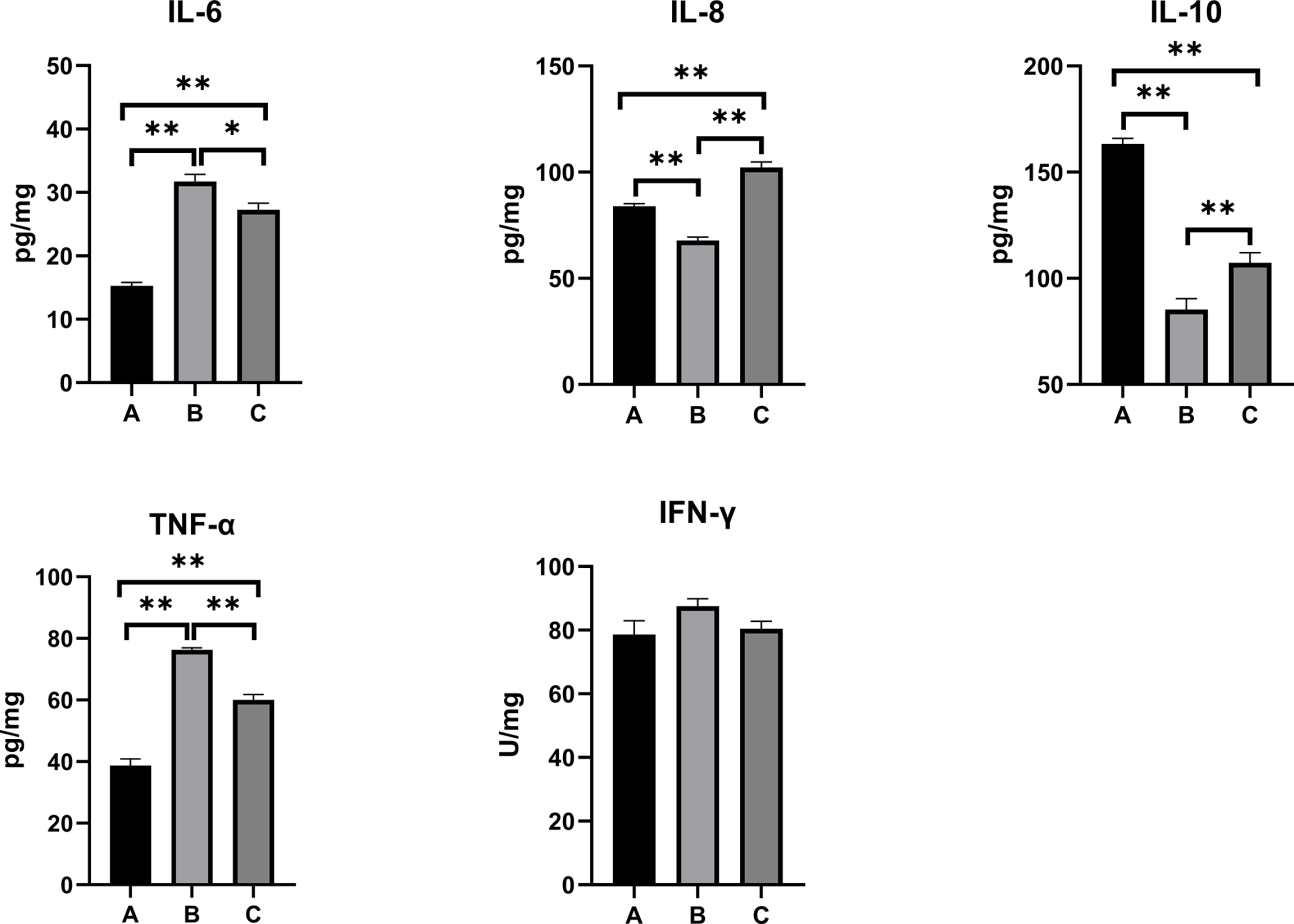
Effects of OTA and AFB1 on liver cytokines (IL-6, IL-8, IL-10, TNF-α, and IFN-γ) of pullets after 42 days (n = 6, mean ± SEM). Groups A, B, and C are the control group, the pair-feeding group, and the OTA+AFB1 group, respectively. Data were analyzed with ANOVA and Tukey’s HSD, and significant differences were defined as *p < 0.05 and **p < 0.01. OTA, ochratoxin A; AFB1, aflatoxin B1; HSD, honestly significant difference.

The inflammatory responses in the pullets’ serum, kidney, and liver are represented in **Figures 5**–**7**. In Group B and Group C, the levels of serum IL-6 significantly increased, while the levels of serum IL-10 significantly decreased when compared with Group A (*p* < 0.01). In Group B, the level of serum IL-8 was significantly lower and the level of serum IFN-γ was significantly higher as compared with other groups (*p* < 0.01). The concentrations of serum IL-8 and TNF-α increased significantly in Group C compared with other groups (*p* < 0.01). In the kidney, the levels of TNF-α decreased significantly in Group B (*p* < 0.05) and Group C (*p* < 0.01) compared with Group A. The level of kidney IFN-γ in Group B was significantly higher as compared with other groups (*p* < 0.01). Moreover, the levels of kidney IL-6 and IL-8 significantly increased, while IL-10 (*p* < 0.01) and IFN-γ (*p* < 0.05) significantly decreased in Group C compared with Group A. When compared with Group A, the levels of liver IL-6 and TNF-α increased significantly, and IL-10 decreased significantly in Group B and Group C (*p* < 0.01). The levels of liver IL-8 in Group C significantly increased compared with other groups (*p* < 0.01).

### Histopathological Findings

**Figure 8:**
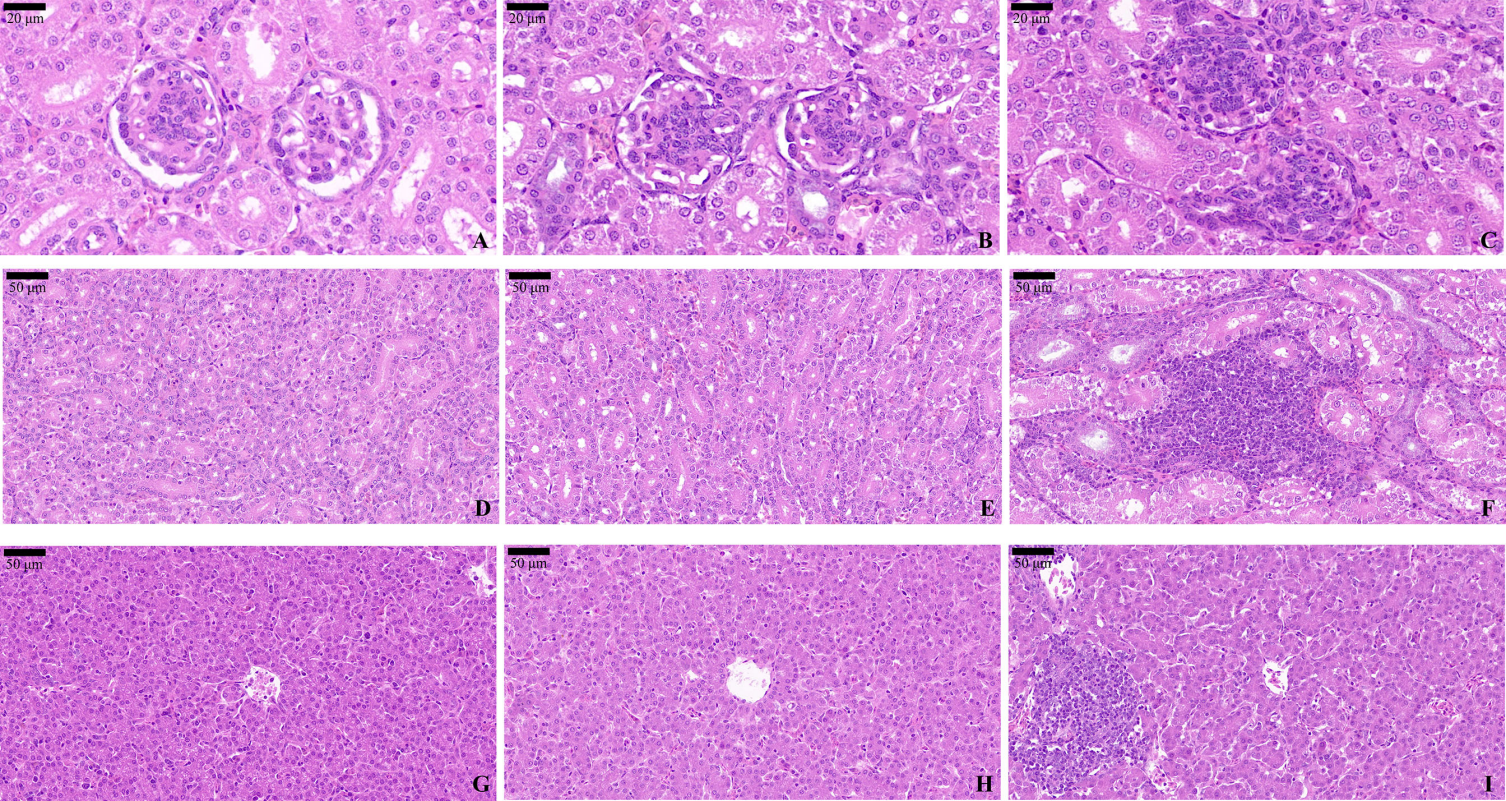
H&E staining of the pullets’ kidney and liver after 42-day trial. Groups A, B, and C are the control group, the pair-feeding group, and the OTA+AFB1 group, respectively. **(A)** Glomeruli of Group A, ×90. **(B)** Glomeruli of Group B, ×90. **(C)** Glomeruli of Group C, ×90. **(D)** Renal tubules of Group A, ×40. **(E)** Renal tubules of Group B, ×40. **(F)** Renal tubules of Group C, ×40. **(G)** Liver of Group A, ×40. **(H)** Liver of Group B, ×40. **(I)** Liver of Group C, ×40. OTA, ochratoxin A; AFB1, aflatoxin B1.

H&E staining analysis was used to evaluate the impacts of OTA and AFB1 on renal and hepatic morphology in pullets. In Group A (**Figure 8A**) and Group B (**Figure 8B**), glomeruli were observed in regular histological structure and with distinct Bowman intervals. The thin and continuous basal membrane of the glomeruli was clearly determined. However, various degrees of glomerular cell hyperplasia were commonly identified in Group C pullets’ kidney sections (**Figure 8C**). Apart from that, it was obvious that Bowman’s space completely disappeared in certain glomeruli. In Group A (**Figure 8D**) and Group B (**Figure 8E**), the basal membranes of the renal tubules were thin and continuous and had a normal histological appearance. In Group C pullets’ kidneys, focal inflammatory cells were common in peritubular interstitial tissues (**Figure 8F**). Inflammatory cell infiltration in the interstitial area, collapsed lumen, and hyaline change were observed in Group C kidney sections.

In the liver sections, a central vein ran through the middle of the hepatic lobules and was surrounded by cords of liver cells that radiated out in all directions in Group A. As for the histological morphology, there was no significant difference between Group A (**Figure 8G**) and Group B (**Figure 8H**). When pullets were fed with combined OTA and AFB1 (Group C), their liver cells displayed unclear line arrangement. Loss of sinusoidal spaces and focal inflammatory cells were also found in the hepatic lobules (**Figure 8I**).

### Apoptosis in Kidney and Liver

**Figure 9:**
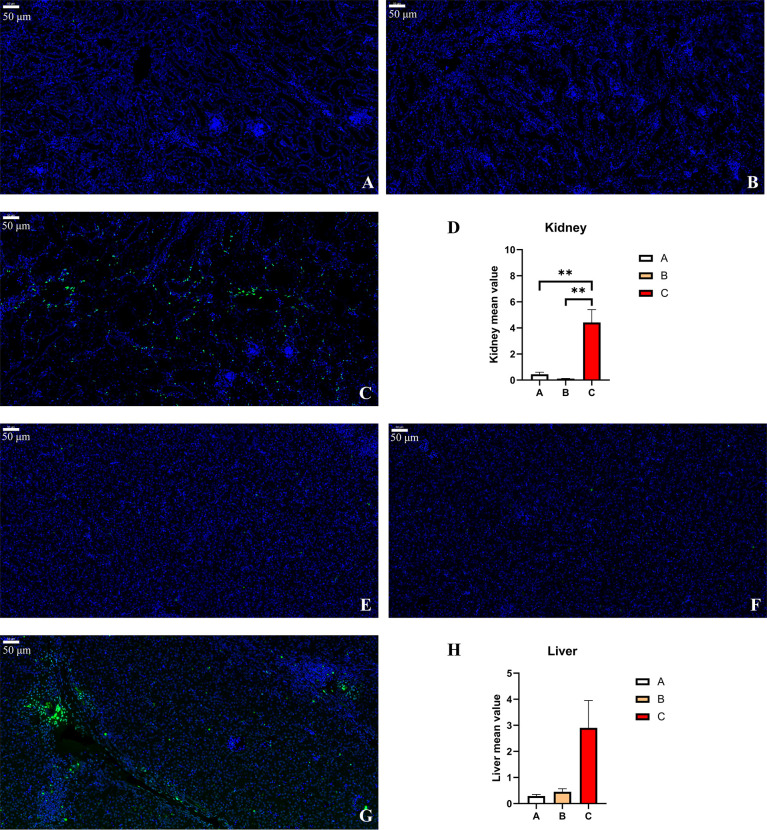
TUNEL staining and fluorometry of the pullets’ kidney and liver after 42-day trial. Groups A, B, and C are the control group, the pair-feeding group, and the OTA+AFB1 group, respectively. **(A)** Kidney of Group A, ×20. **(B)** Kidney of Group B, ×20. **(C)** Kidney of Group C, ×20. **(D)** Detection of caspase-3 enzyme activity by fluorometry in chick kidney (n = 3, mean ± SEM, **p < 0.01). **(E)** Liver of Group A, ×20. **(F)** Liver of Group B, ×20. **(G)** Liver of Group C, ×20. **(H)** Detection of caspase-3 enzyme activity by fluorometry in chick liver (n = 3, mean ± SEM). OTA, ochratoxin A; AFB1, aflatoxin B1.

In order to evaluate the apoptosis in experimental pullets, the kidney and liver sections were stained using the TUNEL method. There were few TUNEL-positive cells in the kidney (**Figures 9A, B**) and liver (**Figures 9E, F**) sections of Group A and Group B. In **Figure 9C**, there were many TUNEL-positive cells scattered around the kidney tissues of Group C pullets, which was confirmed by the significantly higher fluorescence intensity in Group C than in the other groups (*p* < 0.01) (**Figure 9D**). Likewise, it was also clear that a few TUNEL-positive cells could be observed in Group C chick liver (**Figure 9G**). The increased intensity of fluorescence was determined in Group C, albeit there was no statistical significance due to the relatively high difference within Group C (**Figure 9H**). In addition, the fluorescence intensity in the liver was higher as compared to the kidney.

### Effects of Ochratoxin A and Aflatoxin B1 on the Gut Microbial Composition of Pullets

**Figure 10:**
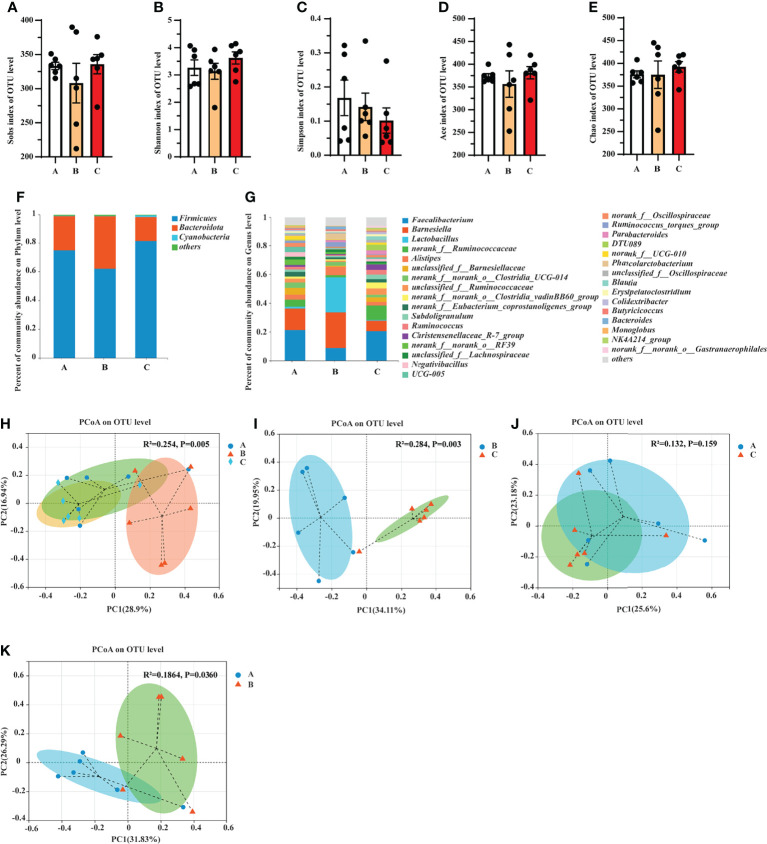
Groups A, B, and C are the control group, the pair-feeding group, and the OTA+AFB1 group, respectively. (A–E) The effects of OTA and AFB1 on the alpha diversity of the gut microbiota in pullets after 42-day trial (n = 6, mean ± SEM). **(A)** Sobs index of the community diversity. **(B)** Shannon index of the community richness. **(C)** Simpson index of the community diversity. **(D)** ACE index of the community diversity. **(E)** Chao index of the community diversity. Data were analyzed with ANOVA and Tukey’s HSD. **(F, G)** The effects of OTA and AFB1 on the relative abundance of the gut microbial community structure in pullets after 42-day trial (n = 6). **(F)** Relative abundance of gut microbiota at the phylum level. **(G)** Relative abundance of gut microbiota at the genus level. (H–K) The effects of OTA and AFB1 on the PCoA (Bray–Curtis distance) plot of the gut microbiota in pullets after 42-day trial (n = 6). **(H)** The PCoA in the three treatments. **(I)** The PCoA in Group B and Group C **(J)** The PCoA in Group A and Group C **(K)** The PCoA in Group A and Group **(B)** OTA, ochratoxin A; AFB1, aflatoxin B1; HSD, honestly significant difference; PCoA, principal coordinate analysis.

16S rDNA sequencing was conducted to assess the influence of OTA and AFB1 on the gut microbial composition in pullets. As a result, 813,801 sequences were acquired. Based on 97% similarity, the sequences were divided into different groups through a clustering operation. Each group was defined as an out; then a total of 667 operational taxonomic units (OTUs) were obtained and divided into 18 phyla, 30 classes, and 189 genera. The results of the alpha diversity analysis showed that there was no significant difference between the Sobs index, Shannon index, Simpson index, ACE index, and Chao index among the three treatment groups (**Figures 10A–E**).

The relative abundance was invested at the phylum and genus levels. At the phylum level, Firmicutes and Bacteroidota accounted for the largest proportion in the cecum of pullets, exceeding 95% of the total cecum bacteria. In addition, the abundance of Firmicutes/Bacteroidetes in Group C was higher than in Group A, while that of Group B was lower than in Group A (**Figure 10F**). At the genus level, *Faecalibacterium*, *Barnesiella*, *Lactobacillus*, and *Ruminococcus* accounted for nearly 40% of the total cecum bacteria (**Figure 10G**). The abundance of *Barnesiella* decreased in Group C and increased in Group B compared with Group A. The proportion of *Faecalibacterium* was similar in Group A and Group C, while it decreased in Group B. It was noteworthy that the abundance of *Lactobacillus* increased dramatically in Group B compared with other groups. The proportion of *Ruminococcus* increased in Group C compared with other groups. Based on the Bray–Curtis distances, a principal coordinate analysis (PCoA) was performed to evaluate the differences in the bacterial community structure of the gut microbiota (**Figure 10H**). The results showed that the gut microbiota of pullets in Group C was altered by the mycotoxins, which was much different from that of Group B (**Figure 10I**). Moreover, the distribution of spots in Group C was more concentrated than in Group A (**Figure 10J**). By reducing the feed supply, a distinguished difference in the composition of microbiota emerged between Group B and Group A (**Figure 10K**).

**Figure 11:**
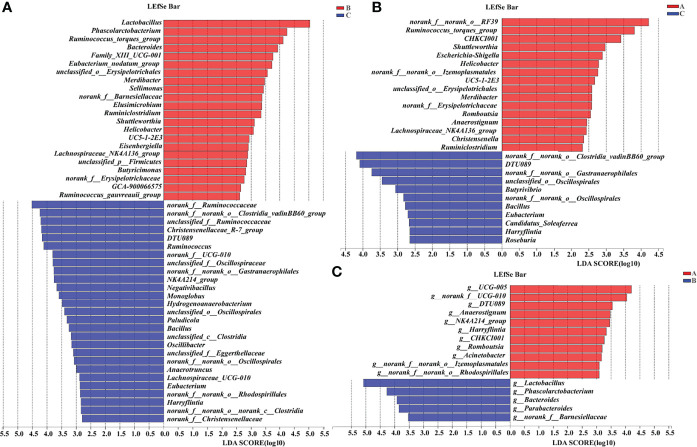
LEfSe analysis of the 16S rRNA sequences of gut microbiota in pullets after 42-day trial (n = 6, LDA > 2). Groups A, B, and C are the control group, the pair-feeding group, and the OTA +AFB1 group, respectively. **(A)** The LEfSe analysis of the gut microbiota in Group B and Group **(C, B)** The LEfSe analysis of the gut microbiota in Group A and Group C **(C)** The LEfSe analysis of the gut microbiota in Group A and Group **(B)** LEfSe, linear discriminant analysis effect size; LDA, linear discriminant analysis.

A supervised comparison of samples was analyzed using the linear discriminant analysis effect size (LEfSe) and logarithmic linear discriminant analysis (LDA). The results showed that in Group C, the gut microbiota was characterized by bacteria *Ruminococcus*, *Clostridium*, *Christensenella*, *Oscillospira*, *Gastranaerophilales*, *Negativibacillus*, *Monoglobus*, *Hydrogenoanaerobacterium*, *Paludicola*, *Bacillus*, *Eggerthella*, *Anaerotruncus*, *Lachnospira*, *Eubacterium*, *Lachnospira*, and *Harryflintia* as compared with Group B (**Figure 11A**). When compared with Group A, Group B was differentially enriched with bacteria *Clostridium*, *Gastranaerophilales*, *Oscillospira*, *Butyrivibrio*, *Bacillus*, *Eubacterium*, *Candidatus Soleaferrea*, *Harryflintia*, and *Roseburia* (**Figure 11B**). Group B was different from Group A in terms of bacteria *Lactobacillus*, *Phascolarctobacterium*, *Bacteroides*, *Parabacteroides*, and *Barnesiella* (**Figure 11C**).

## Discussion

OTA and AFB1 are considered the most notorious contaminants of food and feed, as either individually or a combination of them could lead to health deterioration and economic losses. There is a general agreement that dietary AFs decrease BW, ADG, and ADFI and increase FCR in poultry (32–34). Likewise, studies revealed that OTA adversely affected ADG, ADFI, and laying performance of hens (35). However, the toxicity resulting from the combination of OTA and AFB1 was more severe than when either of these mycotoxins was administrated alone. There was a significant interaction between OTA and AFs on broiler growth performance (36). It has been reported that co-contamination of 56 μg/kg of OTA and 136 μg/kg of AFB1 in feed could significantly decrease ADFI, ADG, egg production, and egg weight in 40-week-old hens (20). In the present study, pullets receiving dietary 101.41 μg/kg of OTA and 20.10 μg/kg of AFB1 had a significant reduction of BW, ADG, and SL and a significant increase of FCR (*p* < 0.01), which was in line with previous studies. The pair-feeding group had significantly lower BW, ADFI, ADG, ADG, and SL as compared with the control (*p* < 0.01). However, the deterioration in pullets’ performance induced by OTA and AFB1 was worse than pair-feeding.

Numerous studies have reported that OTA and AFB1 could affect the relative weight of different organs in animals. Huff (36) reported that a combination of OTA (2.0 mg/kg) and AFs (3.5 mg/kg) in broiler feed could increase the relative weight of the kidneys (1.24 g/100 g BW), liver (4.92 g/100 g BW), heart (0.95 g/100 g BW), and proventriculus (0.95 g/100 g BW) as compared with the control (kidneys 0.56, liver 3.13, heart 0.8, and proventriculus 0.74 g/100 g BW). Furthermore, administration of 56 μg/kg of OTA and 136 μg/kg of AFB1 in 40-week-old laying hens significantly increased the relative weight of the liver (3.12 g/100 g BW), kidneys (0.93 g/100 g BW), heart (0.74 g/100 g BW), and spleen (0.17 g/100 g BW) (*p* < 0.05) (20). The results of the current study showed that OTA and AFB1 significantly reduced the relative weight of the spleen (0.27 g/100 g BW) and significantly increased that of the left kidney (0.53 g/100 g BW) (*p* < 0.01) when administrated dietary 20.10 μg/kg of AFB1 and 101.41 μg/kg of OTA. There was also an uptrend in liver relative weight (3.27 g/100 g BW), although not statistically significant (*p* = 0.09). The relative weight of the kidney and liver was commonly increased by the mycotoxins’ exposure. In the present study, the change in the relative weight of the spleen was different from the mentioned study (20), which probably resulted from the almost double dose of OTA and the much lower dose of AFB1. Chicks are more sensitive to mycotoxins than laying hens, which may also contribute to the different results. Overall, the relative weight of organs may be influenced by different animal species, age, experiment period, and the dose and kinds of mycotoxins. We found that reducing feeding did not influence the relative weight of organs.

Biochemical parameters such as several proteins and metabolites and certain enzymes can be used as sensitive indicators of mycotoxin toxicity. The serum AAP, LAP, PEPCK, and creatine were often used to evaluate *in vivo* kidney damage, whereas ALP, AST, ALT, cholesterol, and triglyceride generally represent impaired liver function (35,37). In the mycotoxin-fed pullets of the current study, the concentrations of serum AAP increased significantly (*p* < 0.01), while PEPCK decreased significantly (*p* < 0.01) as compared with the control. In addition, there was also an uptrend in the serum levels of ALP and AST in Group C compared with the control, though not statistically significant. As for Group B, there were significantly higher levels of AAP and PEPCK than in the control (*p* < 0.01); the level of LAP was significantly lower than in the control (*p* < 0.01). The concentration of serum ALT in Group B was significantly higher than in Group C (*p* < 0.01). Overall, these results indicated potential kidney and liver damage in the pair-feeding and the OTA+AFB1 pullets.

Several studies suggest that mycotoxin exposure leads to the overproduction of free radicals. SOD, CAT, GSH, and GSH-Px are important antioxidants that can prevent oxidative damage induced by ROS (11). It has been reported that the broiler fed with 100 μg/kg of AFB1 had significantly decreased antioxidant enzyme activities of total SOD, CAT, GSH-Px, glutathione S-transferase, and T-AOC but increased MDA content (38). Similar results were demonstrated in OTA-fed (1.6, 3.2, and 6.4 mg/kg of feed) broiler chicks; a significant dose-dependent decrease in the SOD, GSH-Px, and total antioxidant status levels was found in the supernatant of tissue homogenates (liver, kidney, and muscles) (39). However, the present study showed higher levels of CAT and ROS in mycotoxin-fed pullets’ serum, kidney, and liver when compared with the other two groups. The antioxidative responses in the kidney were the most active among these three groups, as there was a significant increase in the levels of SOD, CAT, and GSH (*p* < 0.01); there was a significant decrease in the levels of MDA, GSSG, and GSH-Px (*p* < 0.01). The contradictory results of the parameters might be caused by the interactions between the mycotoxins and the reduction of the feed supply. When the pair-feeding group was compared with the control, the levels of T-AOC decreased in the serum but increased in the liver; the levels of GSH increased in the serum and the kidney but decreased in the liver; the levels of GSSG and GSH-Px decreased in the serum and the kidney but increased in the liver. The possible reason for these results might be that the organism activated most of the antioxidative system to repair the most damaged kidney, while other organs and tissues were not adequately repaired. Overall, a longtime low dose of OTA and AFB1 activated the antioxidative reactions of chicks, which was weakly influenced by the feed intake reduction.

As for inflammation cytokines, the levels of mycotoxin-exposed pullets’ serum, kidney, and liver IL-6 and IL-8 significantly increased and anti-inflammation cytokine IL-10 significantly decreased (*p* < 0.01) as compared with the control group. In Group C, although the concentration of TNF-α significantly decreased in the kidney (*p* < 0.01), it significantly increased in the serum and liver (*p* < 0.01). Likewise, significant increases in the serum levels of TNF-α and IFN-γ were discovered in broiler chickens fed with 74 μg/kg of AFB1 (40). After administration of oral OTA (235 μg/kg BW) in ducklings, higher levels of serum and liver IL-6 and IL-1β and a lower level of IL-10 were found (41). When the pair-feeding group was compared with the control, the levels of serum and liver IL-6 significantly increased (p < 0.01); the concentrations of serum and kidney IL-8 and IL-10 significantly decreased (p < 0.01). Likewise, the levels of serum and the kidney IFN-γ of the pair-feeding group were significantly higher than in the other two groups (*p* < 0.01); the concentrations of TNF-α of the pair-feeding group significantly decreased in the kidney (*p* < 0.05) but increased in the liver compared with the control (*p* < 0.01). Taken together, OTA+AFB1 induced the inflammatory responses in the pullets, while the reduction of the feed intake influenced it.

Apart from cytokines, focal inflammatory cells and apoptosis cells were observed in the kidney and liver sections of OTA- and AFB1-exposed pullets, while those in the pair-feeding group and the control remained relatively normal. It has been reported that the livers of AFB1-supplemented rabbits showed fatty degeneration with vacuolization, focal areas of necrosis, mononuclear cell infiltration hyperplasia of bile ducts, and sinusoids (42). There were multifocal inflammation, hepatocellular vacuolation, and bile duct hyperplasia in the liver tissues of chicks supplemented with 0.1 mg/kg of AFB1 and 1 mg/kg of OTA. Moreover, glomerulonephritis alongside focal necrosis, vacuolar degeneration, and vacuolar generation findings was also noted (43). There was a study that demonstrated that numerous TUNEL-positive apoptotic bodies could be determined in the area of focal necrosis. Moreover, some TUNEL-positive mononuclear cells were scattered in liver parenchyma and portal tracts. In chronic hepatitis, some hepatocytes in the area of piecemeal necrosis possessed TUNEL-positive nuclei. In the fibrously enlarged portal area, TUNEL-positive mononuclear cells often were infiltrated (44). Taken together, the administration of OTA and AFB1 on pullets leads to high levels of cytokines, histopathological changes, and apoptosis in the kidney and liver.

The gut is the first target of OTA and AFB1 in the feed that enters the body, and it is tightly associated with other tissues through enterohepatic circulation. OTA and AFB1 mainly alter the composition to change the gut microbiota, which can occur at the phylum, genus, and species levels (45). Researchers have reported that Firmicutes, Bacteroidetes, and *Ruminococcus* are essential for the maintenance of gut health. For instance, Firmicutes is involved in the energy metabolism (46); Bacteroidetes is involved in the bile acid metabolism and transformation of the toxic compound through the secretion of butyric acid (47); *Ruminococcus* can inhibit the growth of pathogenic microorganisms by producing volatile fatty acids and lactic acid to reduce gut pH (48). The co-exposure of OTA and AFB1 increased the percentage of Ruminococcaceae, Bacteroidaceae, and Lachnospiraceae *in vitro* digestion models (49), which was partly in line with the present study. In the previous studies, *Ruminococcus*, *Bacteroides*, Dorea, *Escherichia*, *Oribacterium*, and *Syntrophococcus* significantly decreased in OTA-treated (70 or 210 μg/kg BW) rats after 28 days of gavage (*p* < 0.01), whereas the increase in the relative abundance of *Lactobacillus* was particularly striking (*p* < 0.01) (50). Wang (51) reported that AFB1 significantly increased Clostridiales of Firmicutes and *Bacteroidales* of Bacteroidetes in rats, while the 2 Lactobacillales from Firmicutes, *Streptococcus* sp., and *Lactococcus* sp. decreased. At the genus level, *Faecalibacterium*, *Barnesiella*, *Lactobacillus*, and *Ruminococcus* accounted for nearly 40% of the total cecum bacteria in the present study. The results showed that the abundance of Firmicutes/Bacteroidetes and *Ruminococcus* increased in Group C compared with other groups, which deduced that pullets could ameliorate the toxic effects caused by OTA and AFB1 by increasing the abundance of gut Firmicutes and *Ruminococcus*. In addition, the gut microbiota of the OTA+AFB1 pullets was characterized to be enriched by the bacteria *Ruminococcus*, *Clostridium*, *Christensenella*, and *Oscillospira*. It was reported that *Ruminococcus gnavus* is a mucin degrader (52) and linked to inflammatory bowel disease (IBD) (53); the level of Clostridiales was strongly correlated with IBD severity (54). *Clostridium*, such as *Clostridium tetani*, *Clostridium botulinum*, and *Clostridium perfringens*, can produce exotoxin and is related to several diseases (55). Breton et al. (56) found that starvation can upregulate several proteins synthesized by the order Clostridiales including Clostridiaceae of mice. As for *Christensenella*, *Christensenella minuta* demonstrated a strong anti-inflammatory activity, resulting in reduced levels of pro-inflammatory IL-8 cytokines *via* the inhibition of the NF-κB signaling pathway (57). Studies demonstrated that bacteria *Ruminococcus lactari* portrayed a negative correlation with IL-8; *Clostridium* positively associates with the pro-inflammatory monocyte chemoattractant protein-1 (MCP-1), while *Oscillospira* and *Lactobacillus* negatively correlate with MCP-1 (58). These results suggested that the combination of OTA and AFB1 altered the composition of gut microbiota and stimulated the growth of pernicious bacteria to cause toxic effects. Among them, the microbiota related to anti-inflammatory responses was the most influenced. The changes varied strongly with the animal species as well as the dose and duration of mycotoxins.

Moreover, the abundance of *Lactobacillus* increased dramatically in the pair-feeding group compared with other groups. It is widely acknowledged that *Lactobacillus* is essential for maintaining health as well as preventing and treating disease (59,60), so the proper reduction of the feed supply is likely to improve the physical health of pullets. This inference was verified by the characterized enrichment of beneficial bacteria, including *Lactobacillus*, *Phascolarctobacterium*, *Bacteroides*, *Parabacteroides*, and *Barnesiella*.

## Conclusions

Compared with the control group, co-contamination of OTA and AFB1 in feed could adversely affect the growth performance (ADFI, BW, ADG, FCR, and SL), decrease the relative weight of the spleen (*p* < 0.01), and increase the relative weight of the kidney. Apart from that, OTA and AFB1 also activated the antioxidative and inflammation reactions of chicks, increasing the levels of CAT, ROS, and IL-8 as well as decreasing the levels of IL-10, which was weakly influenced by the feed intake reduction. In addition, OTA and AFB1 induced histopathological changes and apoptosis in the kidney and liver as well as stimulated the growth of pernicious bacteria to cause toxic effects. The combined toxicity of OTA and AFB1 had more severe effects on pullets than merely reducing feed supply. However, the proper reduction of the feed intake could improve pullets’ physical health by enriching bacteria *Lactobacillus*, *Phascolarctobacterium*, *Bacteroides*, *Parabacteroides*, and *Barnesiella*.

## Data Availability Statement

The raw data supporting the conclusions of this article will be made available by the authors, without undue reservation. The data presented in the study are deposited in the NCBI repository and accession numbers can be found below: https://www.ncbi.nlm.nih.gov/sra/PRJNA862454.

## Ethics Statement

The animal study was reviewed and approved by Laboratory Animal Welfare and Animal Experimental Ethical Committee of China Agricultural University (No. AW 13301202-1-12).

## Author Contributions

Conceptualization and methodology: HQ and QM. Formal analysis and data curation: HQ and SH. Writing—original draft preparation: HQ. Writing—review and editing: HQ, QM, KZ, LZ, JZ, and CJ. Validation and supervision: SH, KZ, LZ, and QM. Project administration and funding acquisition: QM. All authors have read and agreed to the published version of the manuscript.

## Funding

This research was funded by Beijing Municipal Science Foundation (No. 6172017), Anhui Province Key Laboratory of Livestock and Poultry Product Safety Engineering (No. XM2004), and China Agricultural Research System program (CARS-40-K08).

## Conflict of Interest

The authors declare that the research was conducted in the absence of any commercial or financial relationships that could be construed as a potential conflict of interest.

## Publisher’s Note

All claims expressed in this article are solely those of the authors and do not necessarily represent those of their affiliated organizations, or those of the publisher, the editors and the reviewers. Any product that may be evaluated in this article, or claim that may be made by its manufacturer, is not guaranteed or endorsed by the publisher.
