# Supplementary material for: Combined Toxicity Evaluation of Ochratoxin A and Aflatoxin B1 on Kidney and Liver Injury, Immune Inflammation, and Gut Microbiota Alteration Through Pair-Feeding Pullet Model
Source: Front Immunol. 2022 Jul 29;13:920147. doi: 10.3389/fimmu.2022.920147 (PMC9373725; doi:10.3389/fimmu.2022.920147)
Supplement: Supplementary file 1 [file DataSheet_1.pdf]

Supplementary Table 1. Composition and nutrient levels of diets (% DM).

Item	Basal Diet	Contaminated Diet
Ingredients		
Normal maize	65.00	0
Contaminated maize[1]	0	65.00
Soybean meal (43% CP)	29.50	29.50
Calcium hydrophosphate (21% Ca, 16% P)	1.90	1.90
Limestone (38% Ca)	1.30	1.30
Sodium chloride	0.30	0.30
Soybean oil	1.00	1.00
Zeolite powder	0.26	0.26
Mineral premix[2]	0.30	0.30
Vitamin premix[3]	0.04	0.04
50% Choline chloride	0.10	0.10
78.5% L-lysine-HCl	0.11	0.11
DL-Methionine	0.19	0.19
Total	100	100
Nutritional levels (%)[4]		
Crude protein	18.00	18.03
ME (MJ/kg)	12.11	12.11
Calcium	1.00	1.00
Total Phophorus	0.64	0.64
Non- phytate phosphorus	0.45	0.45
Methionine	0.45	0.45
Methionine + Cystine	0.74	0.74
Lysine	1.00	1.00
Tryptophan	0.20	0.20
Threonine	0.68	0.68

[1] When replacing the normal maize with contaminated maize at 65%, the contaminated diet contains 101.41 µg/kg OTA and 20.10 µg/kg AFB1. [2] Provided per kilogram of diet: vitamin A, 11,700 IU; vitamin D3 3,600 IU; vitamin E, 21 IU; vitamin K3, 4.2 mg; thiamine, 3 mg; riboflavin, 10.2 mg; folic acid, 0.9 mg; calcium pantothenate, 15 mg; nicotinic acid, 45 mg; pyridoxine, 5.4 mg; vitamin B12, 24 µg; biotin, 150 µg. [3] Provided per kilogram of diet: Cu, 6.8 mg; Fe, 66 mg; Zn, 83 mg; Mn, 80 mg; I, 1 mg, Se 0.3 mg. [4] The nutrient levels were calculated values.
